# Study on Electrically Modulated Quasi-Continuous Wave Fe: ZnSe Solid-State Laser with Hundred-Hertz

**DOI:** 10.3390/mi14122194

**Published:** 2023-11-30

**Authors:** Yingchao Wan, Yanlong Shen, Ke Wang, Tongxing Chai, Yousheng Wang, Zhengge Chen, Feng Zhu

**Affiliations:** State Key Laboratory of Laser Interaction with Matter, Northwest Institute of Nuclear Technology, Xi’an 710024, China; wanyingchao18@nudt.edu.cn (Y.W.);

**Keywords:** MIR laser, Fe: ZnSe crystal, quasi-CW, electrical modulation

## Abstract

Iron-doped binary chalcogenide crystals are very promising for tunable solid-state lasers operating over the 3~5 μm spectral range. Fe: ZnSe is one of the most important gain crystals with the obvious advantages of material characteristics and conversion efficiency. By adjusting the output mode of the pump source, an Fe: ZnSe laser can operate in two modes at liquid nitrogen temperatures: continuous wave (CW) and pulse output. In terms of CW output, the Fe: ZnSe laser obtained a maximum 2.63 W continuous power output which was confined to the power of the pump source. An optical-to-optical efficiency of 47.05% was acquired. Direct electrical modulation was applied to the pump source. The highest average power of the quasi-CW laser, whose central wavelength is 4.02 μm, has a value of 253 mW with an optical-to-optical efficiency of 42.88% and a full width at half maximum (FWHM) of 23 nm when the pulse frequency is 100 Hz of 10% duty factor. The output waveform is consistent with the modulation waveform applied to the pump source. We report to the first of our knowledge an electrically modulated quasi-CW Fe: ZnSe laser in the pulse regime, equipped with features of compactness in structure, ignoring additional modulators, convenience in control, high efficiency, and sustainable operation, of great interest for solving numerous scientific and applied problems.

## 1. Introduction

Middle infrared (MIR), with a wavelength of 3~5 μm, is an atmospheric window with a wide range of possible applications in atmospheric remote sensing, medical treatment, industrial processing, infrared countermeasures, space communication, martial utilization, diagnosis of exhaust emission, and other fields [1,2,3,4]. Nowadays, two techniques have attracted interest for generating MIR, including stimulating gain crystals to radiate emission and nonlinear frequency conversion [5,6,7,8,9]. In 1996, DeLoach from Lawerence Livermore Laboratory demonstrated that transition metal (TM)-doped II–VI group sulfide crystals could be used as the gain medium to achieve MIR laser output with huge potential and advantages [10]. Ion Fe^2+^ (TM)-doped II–VI group sulfide crystals are advantageous for their large absorption, emission section, and wide gain linewidth. Additionally, the undoped host ZnSe polycrystal has a wide transmission spectrum in the infrared band with good thermal conduction [11,12,13]. Therefore, great progress has recently been made in solid MIR lasers based on Fe: ZnSe crystals as a gain medium that are directly pumped.

Fe: ZnSe crystals are sensitive to temperature, leading to a temperature-quenching effect at higher temperatures. The lifetime of the laser’s upper level sharply diminishes with temperature acceleration, from dozens of seconds at 77 K cooled by liquid nitrogen to 370 ns at room temperature (294 K) [14]. Thus, a liquid nitrogen temperature controller promises continuous operation of Fe: ZnSe lasers. At room temperature, only a narrow pulse source can be used to generate efficient resonance. In 2008, Voronov from Russia reported a Fe: ZnSe laser that was pumped by a CW source for the first time. The highest power obtained was 160 mW with a slope efficiency of 56% [15]. In 2017, Martyshkin from IPG in America generated a 9.2 W laser using Fe: ZnSe with a Cr: ZnSe CW laser as the pump source, whose power is currently the highest reported. The slope efficiency was 41.2% [16]. As for Fe: ZnSe pulse lasers, the characteristics of an Fe: ZnSe laser were studied at the temperature of 85 K based on Er: YAG laser excitation. The highest output energy value was 10.6 J. The threshold-absorbed pump energy was as low as 60 mJ, calculating a conversion of 37% [17]. The highest energy was 1.67 J pumped by an HF laser at room temperature [18].

A quasi-CW laser is defined as a pulse laser with a pulse width in the magnitude of ms, with features of low repetition frequency (from hundreds of Hz to kHz), large duty ratio (the percentage of one period in which a signal is active) (reaching 10%), high power, strong energy, and combining the outputs of CW and pulse. In this present work, direct electrical modulation was applied to the pump source, exciting the Fe: ZnSe crystal. The extra modulator was not in demand, making the configuration compact and convenient to control, and we were able to freely switch the output between CW and pulse. We demonstrated a MIR laser with CW or pulse output when the temperature of the Fe: ZnSe crystal was controlled by liquid nitrogen. Meanwhile, the frequency of the modulation signal was 100 Hz with a duty ratio of 10%. The quasi-CW laser was found to have the highest average power of 253 mW, whose central wavelength peaked at 4.02 μm with an FWHM of 23 nm and optical-to-optical efficiency of 42.88%. The different methods for pumping, forward and backward, were also studied to make a comparison and determine their influence on promoting the performance of the Fe: ZnSe laser.

## 2. Materials and Methods

**Figure 1:**
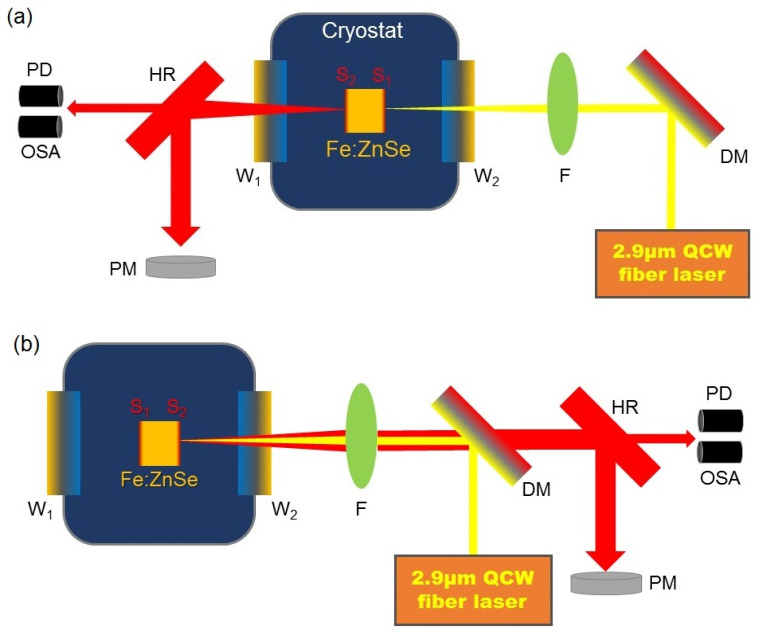
Experimental schematic diagram of Fe: ZnSe laser. (**a**) Forward pump; (**b**) backward pump. PD: photoelectric detector; OSA: optical spectral analyzer; PM: power meter; F: focusing lens; DM: dichroic mirror.

Figure 1a shows the scheme of the Fe: ZnSe laser excited by a forward pump structure. Pump light was filtered by a dichroic mirror (DM, HR > 99%@2.6~3 μm, HT > 95%@3.7~4.8 μm, angle of incidence at 45°) after collimation. Then, the light was focused on the Fe: ZnSe crystal which was fixed in the cryostat and wrapped with indium foil. The focal length of the focus was 50 mm. After completing the energy conversion in the crystal, the excited laser was detected through a high-reflection mirror with a power meter (PM), optical spectral analyzer (OSA), and photoelectric detector (PD). Corresponding parameters were obtained. The pump source was a self-developed electrical modulation quasi-CW fiber laser with a central wavelength of 2.9 μm. The gain medium of this quasi-CW fiber laser is a Ho^3+^, Pr^3+^ co-doped ZBLAN fiber of about 6 m in length, and the pump source is a laser diode (LD) with a central wavelength of 1150 nm. In the CW regime, the maximum output of the pump source was 5.6 W. When the repetition frequency was 100 Hz with a duty ratio of 10%, a square signal with an average power of 590 mW. The parameters of the pulse were adjusted via tuning modulation applied to the pump LD such as repetition frequency, duty ratio, and waveform. The pulse waveform of the output laser is highly identical to the waveform of the pulse electrical current loaded on the LD. In this report, a technique for overshoot pulse modulation was not considered.

The Fe: ZnSe crystal served as the gain medium with dimensions of 7.2 × 7.2 × 9 mm^3^. The concentration of ion Fe was 5 × 10^18^ cm^−3^. Due to the low rigidity of the crystal, scratches easily appeared. Indium foil was chosen to protect the crystal from damage. The bulk of the crystal was installed in the heat sink, avoiding heat accumulation on the crystal and decreasing the lifetime of Fe^2+^. A liquid nitrogen temperature controller was employed with the CaF_2_ mirror as a window, with an anti-reflection membrane at 2.7~4.8 μm. To make the output laser steady, the Fe: ZnSe crystal was polished and coated. The front face, S1, showed high transmittance of the pump light (HT ≥ 95%@2.6~3 μm) while showing strong reflection of emission (HR ≥ 99%@3.7~4.8 μm). The other face, S2, was partly transmitted to emission (T~65%@3.7~4.8 μm). The ends of the crystal formed a resonant cavity, promising compactness, a convenient process of tuning the cavity.

**Figure 2:**
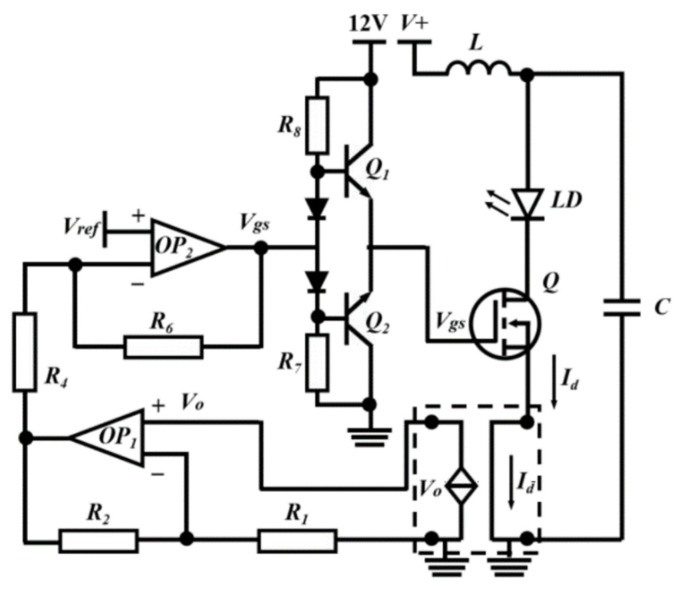
Schematic diagram of the LD drive circuit [19].

The driver circuit of the LD is shown in Figure 2. The in-phase terminal of the differential op-circuit (OP_2_) is connected to the reference voltage (*V_ref_*). The Hall current sensor (part of the frame) senses the magnitude of the drain current (*I_d_*) of the field effect tube (FET) and then outputs the corresponding feedback voltage (*V_0_*), which acts on the inverted end of the differential operation circuit through the series feedback circuit (OP_1_) and the voltage divider resistors *R_4_* and *R_6_*. OP_2_ adjusts the field effect tube gate-source voltage (*V_gs_*) by comparing the magnitude of V_ref_ and V_0_. Since the FET operates in the constant current region, it is possible to indirectly control *I_d_* by controlling *V_ref_*. In this circuit, when *V_ref_* is a continuous signal, the current through the LD is continuous; when *V_ref_* is a pulsed signal, the current through the LD is pulsed. Continuous or pulsed *V_ref_* can be generated by an arbitrary waveform generator (AWG).

## 3. Results

### 3.1. Results of CW Output

**Figure 3:**
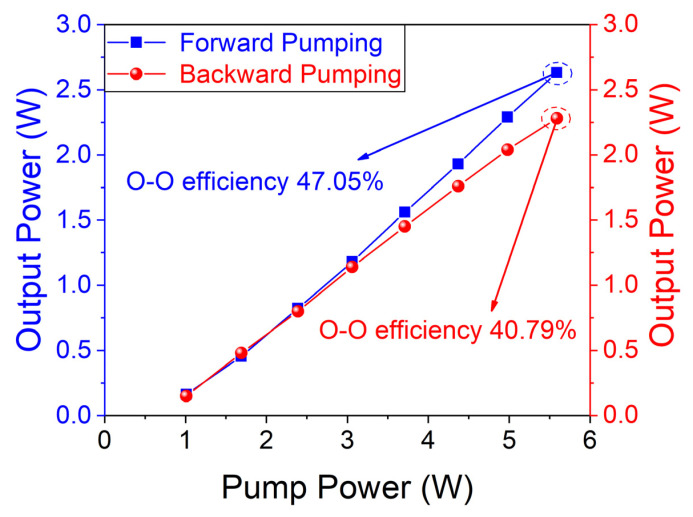
Curves of Fe: ZnSe laser output in CW regime.

The characteristics of the laser were tested in the CW regime, including the output power of different pump structures shown in Figure 3. The blue square represents power when the forward pump is used, while the red spot represents power excited by the backward pump. As the power of the pump source increases, the output power is enhanced without a trend of saturation. The two lines grow linearly until the peak value. The highest output power of the blue line is 2.63 W with an efficiency of 47.06%. As for the red line, the value of output power has a maximum of 2.28 W and an efficiency of 40.79%. The efficiency is defined as the ratio of power measured by PM and quasi-CW fiber laser output power. Comparing the two lines, it is obvious that the forward pump is more advantageous than the backward pump as the perspective of output power when pump power is over 3 W. There is no clear distinction between these two lines excited by the low pump. The reason why the difference is caused, we assume, is that two factors contribute to the result. The pump light enters the crystal through the front face, S_1_, which is HR-coated at 2.6~3 μm. Less pump energy is lost when a forward pump is built. On the contrary, in the structure of the backward pump, as shown in Figure 1b, the output laser has more loss, propagating through one more focusing lens and the DM. The ultimate total output power is lower.

**Figure 4:**
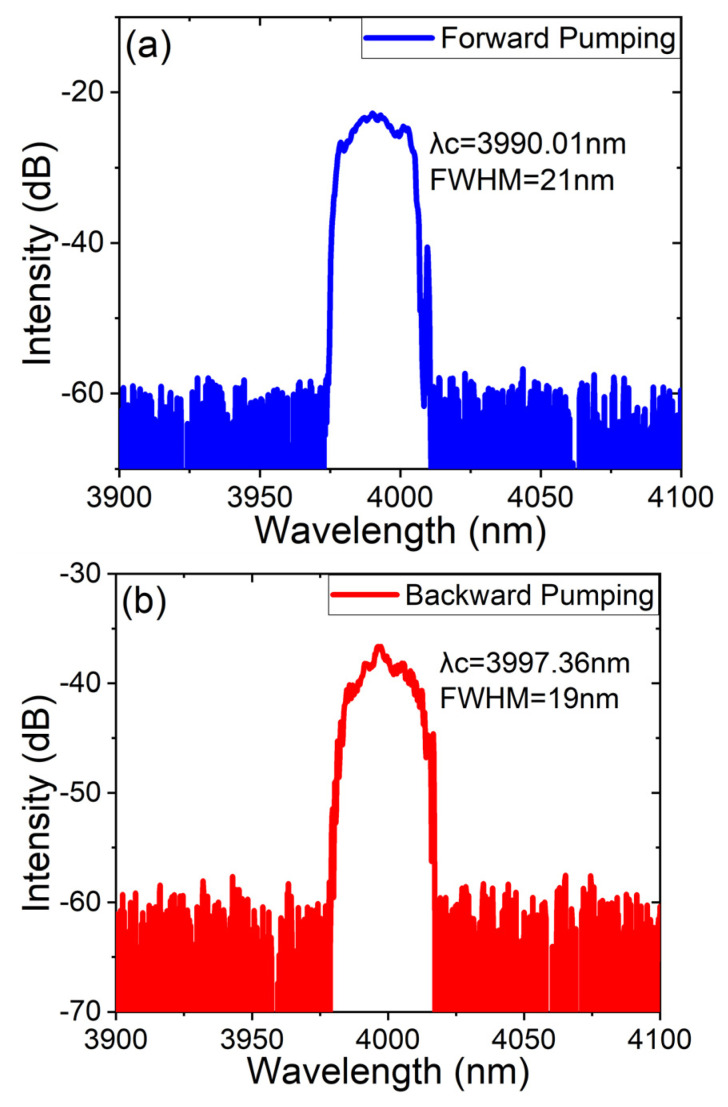
Spectra of Fe: ZnSe laser in CW regime. (**a**) Forward pump; (**b**) backward pump.

Not only was power measured, but the spectra were analyzed, as shown in Figure 4. In Figure 4a, the time of the highest output power excited by the forward pump is drawn using OSA. Similarly, Figure 4b shows the spectrum acquired from the backward pump. From the spectra, it can be seen that the wavelength of the laser output is centered at 4 μm, consistent with the emission spectrum of the Fe: ZnSe crystal. The results show that conversion between wavelengths occurred. The central wavelength is 3990.01 nm with an FWHM of 21 nm in the forward structure while the central wavelength is 3997.36 nm with an FWHM of 19 nm in the backward structure.

**Figure 5:**
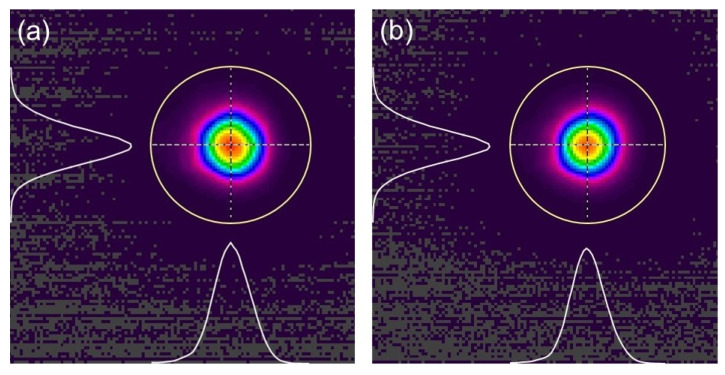
Intensity distribution of Fe: ZnSe laser beam. (**a**) Forward pump; (**b**) backward pump.

At the same time, we recorded the beam profile of the laser via MIR charge-coupled devices (CCD), as shown in Figure 5. The data were detected when output power was at maximum. Figure 5a shows the intensity distribution of the Fe: ZnSe laser beam when the forward pump is utilized. As for the backward pump, Figure 5b shows the result. The beam quality is good, which can be approximately regarded as the TEM_00_ mode. It can be seen that under the structure of the two pump methods, the beam quality demonstrates good performance, which can be approximately regarded as the TEM_00_ mode from the figures. In addition, the energy of the optical spot is in Gaussian distribution.

### 3.2. Analysis of Quasi-CW output

**Figure 6:**
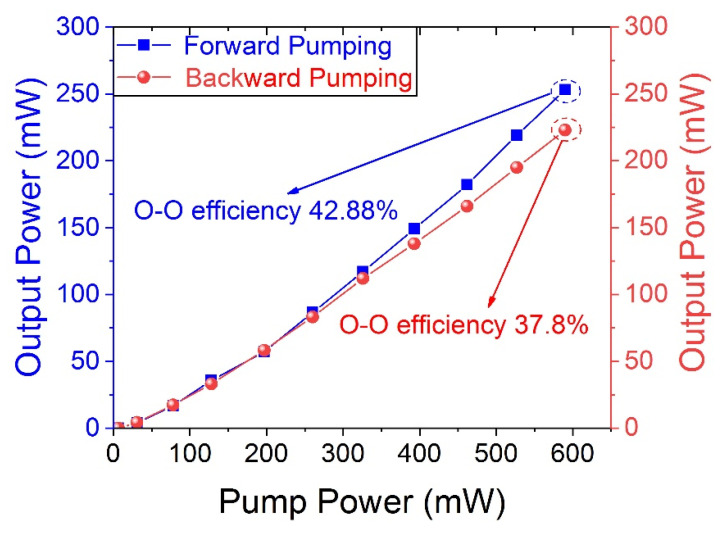
Characteristics of Fe: ZnSe laser when electrical modulation is used.

Figure 6 shows the characteristics of the Fe: ZnSe laser when electrical modulation was used. The frequency is 100 Hz with a duty ratio of 10%. The average power is monitored by PM in the two structures mentioned above, differing between the blue and red lines. The lasing threshold is very low—at 31 mW of pump radiation. The input–output characteristic is nearly fitted by linear dependence with the highest conversion efficiency of 42.88% in the forward pump and 37.8% in the backward pump. The peak power is 253 mW in the forward pump and 223 mW in the backward pump. Compared with CW output, the quasi-CW the same qualitative feature. The structure of the forward pump shows greater power and efficiency. The efficiency of the quasi-CW output is lower than that of the CW output.

**Figure 7:**
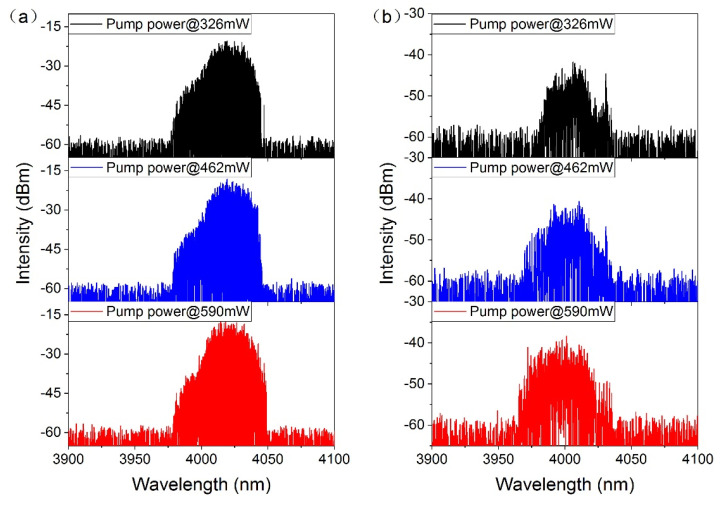
Spectra pumped by different power levels. (**a**) Forward pump; (**b**) backward pump.

Spectra are also depicted in Figure 7 at different pump powers. Figure 7a shows the results of the forward pump while Figure 7b shows the backward pump. As with the CW output, the central wavelength still peaked at ~4 μm. The wavelength of 2.9 μm is transferred to the laser of MIR. As pump power increases, the spectrum widens. When pump power is the highest, the central wavelength is 4020.71 nm with 23 nm FWHM in the forward pump, and 3992.15 nm with 40 nm FWHM in the backward pump.

**Figure 8:**
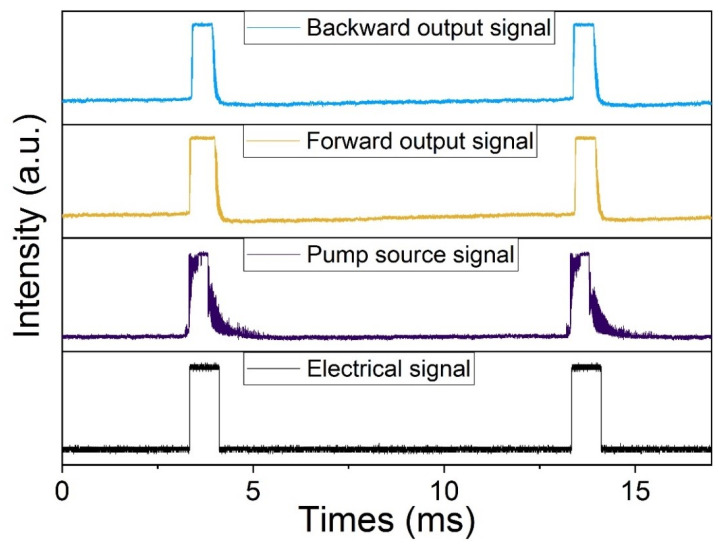
The waveform of modulation signal and laser output.

The waveforms of different signals are given in Figure 8. The black line is the modulation signal which is a square wave with a frequency of 100 Hz, duration ratio of 10%, and 1 ms pulse width. The purple line shows the pump waveform at the maximum output. The yellow line represents the waveform of the Fe: ZnSe laser in the forward structure. The parameters are measured as a frequency of 99.1 Hz, pulse width of 0.78 ms, and energy of 2.55 mJ. The blue line indicates the waveform of the Fe: ZnSe laser in the backward structure. The parameters are measured as a frequency of 100.3 Hz, pulse width of 0.67 ms, and energy of 2.22 mJ. It can be seen that whatever structure is fabricated, the Fe: ZnSe laser can maintain a square wave output with a frequency of around 100 Hz.

## 4. Discussion and Conclusions

The conclusion can be drawn that an Fe: ZnSe laser with a wavelength of 4 μm was built by directly loading electrical modulation on a pump LD. High optical-to-optical efficiency is obtained, and there was no need to make use of other modulators. The scheme serves as one of the most important techniques to realize output with strong pulse energy and high peak power. In our study, restrained by the power of the pump source and frequency of modulation, a limitation of laser power was identified. In the future, we plan to improve the performance of the Fe: ZnSe laser in four aspects. On the one hand, the theoretical analysis needs to be strengthened, combined with the experimental results. The parameters of the Fe: ZnSe crystal and resonator membrane will be optimized to promote conversion efficiency. On the other hand, the “overshoot pulse modulation” technique may be in demand to replace the original and ordinary pump, making it possible to enhance the output of strong energy and high power. In addition, continuously improving pump power to accomplish calibrated amplification of the output power on the existing foundation. Ultimately, MIR diffraction grating can be used to build a resonator instead of a reflective film. The output wavelength can be tuned because of the advantage of the Fe: ZnSe crystal having the characteristic of range emission spectra spanning from 3.7 μm to 5.1 μm, with tunable MIR output being achieved.

In summary, we have presented an electrically modulated quasi-CW Fe: ZnSe laser for the first time. The emission wavelength is centered at 4.02 μm with 23 nm FWHM. Not only was the CW generated at the maximum power of 2.63 W, but the quasi-CW laser could be excited. A square wave pulse was directly loaded on the pump LD. The highest average power was acquired at 253 mW with an optical-to-optical efficiency of 42.88% in the forward pumping configuration. The output energy of the Fe: ZnSe laser was 2.55 mJ with a pulse frequency of 99.1 Hz, whose pulse width was measured as 0.78 ms. The waveform of the laser and pump light was almost identical to the electrically modulated signal. The Fe: ZnSe laser introduced in this paper can operate both in CW and quasi-CW regimes with a compact structure, high slope efficiency, and dispensing with extra modulators. The technique we study provides a basis for further miniaturized and wavelength-tunable Fe: ZnSe lasers equipped with strong pulse energy and high peak power, with many significant potential applications in mid-infrared fields such as environmental monitoring, laser communication, remote sensors, and national defense.

## Data Availability

The data presented in this study are available on request from the corresponding author.
